# 

**Published:** 1901-09

**Authors:** 


### Society Notes.

EASTERLY, TEXAS, August 31, 1901.

The 12th semi-annual meeting of the Brazos Valley Medical Association will be held in the city of Bryan on the second Tuesday and Wednesday in November next. A first class program is promised. A full attendance is desired.

DANIEL PARKER, President.

W. B. BRIGGS, Secretary.

---

The Southwestern Tri-State Medical Society (Texas, Oklahoma and Indian Territory) will meet in Dallas on October 1, 1901. This society was organized last year and it is believed has a very promising future.

All communications relating to the reading of papers and other matters should be sent to Dr. S. E. Milliken, Secretary.

H. K. LEAKE, President.

---

The 13th annual meeting of the Tri-State Medical Society will be held at the Tulane, Nashville, Tuesday, Wednesday and Thursday, October 8, 9 and 10, 1901. The attendance promises to be large, and an unusually attractive program will be presented. The railroads will give reduced rates.

Those intending to read papers should send titles to Dr. Frank Trester Smith, Chattanooga.

---

The Health Officers' Association of Texas met in Dallas August 6th. There was a slim attendance owing to misunderstanding of date. The next meeting will be held in Austin, 2nd Tuesday in January. Neither the president nor secretary were present. Officers elected were president, Dr. I. J. Jones, of Austin; Dr. J. E. Wilson, Dallas, Secretary.

